# Access and Interest: Two Important Issues in Considering the Feasibility of Web-Assisted Tobacco Interventions

**DOI:** 10.2196/jmir.1000

**Published:** 2008-11-03

**Authors:** John A Cunningham

**Affiliations:** ^1^Centre for Addiction and Mental Health and University of TorontoTorontoONCanada

**Keywords:** Internet access, Internet availability, cigarettes, brief interventions, self-help, epidemiological survey

## Abstract

**Background:**

Previous research has found that current smokers are less likely to have access to the Internet than nonsmokers. As access to the Internet continues to expand, does this finding remain true? Also, how many smokers are interested in Web-assisted tobacco interventions (WATIs)? These questions are important to determine the potential role that WATIs might play in promoting tobacco cessation.

**Objectives:**

The aims of the study were to determine whether smokers are less likely than nonsmokers to have access to the Internet and to establish the level of interest in WATIs among a representative sample of smokers.

**Methods:**

A random digit dialing telephone survey was conducted of 8467 adult respondents, 18 years and older, in Ontario, Canada from September 2006 to August 2007. All respondents were asked their smoking status and whether they used the Internet (at home or work in the past 12 months; where; how often in the past 12 months). To assess the level of interest in WATIs, current daily smokers were asked whether they would be interested in a confidential program that they could access on the Internet, free of charge, that would allow them to check their smoking and compare it to other Canadians.

**Results:**

Smokers were marginally less likely to have used the Internet than nonsmokers (74% vs 81% in the last year), and, of those who had access to the Internet, smokers used the Internet less often than nonsmokers. Overall, 40% of smokers said they would be interested in a WATI. The number of cigarettes smoked per day was unrelated to level of interest in the WATI, but time to first cigarette after waking was. Smokers who used the Internet were more interested in the WATI than smokers who did not use the Internet (46% vs 20%).

**Conclusions:**

While the difference in level of Internet use between smokers and nonsmokers was greatly reduced compared to 2002 and 2004 data, smokers still remain marginally less likely to use the Internet than nonsmokers. Overall, there was a substantial level of interest in the WATI among smokers, in particular among smokers who currently use the Internet. These results indicate that WATIs have a substantial potential audience among smokers, and, given the growing body of evidence regarding their efficacy, there is growing support that WATIs have a significant role to play in promoting tobacco cessation.

## Introduction

There are at least three issues of importance when considering whether Web-assisted tobacco interventions (WATIs) are a feasible way to help large numbers of people quit smoking. The first of these issues is the efficacy of the interventions. Do WATIs work? There is a growing body of research indicating that WATIs are an effective means of promoting tobacco cessation. Most notably, there have been several randomized controlled trials to date that have found that WATIs increase the rate of successful cessation from smoking cigarettes (eg, [1-3]).

The other two issues of importance are whether smokers can easily access WATIs and whether they are interested in doing so. These topics are the focus of this paper. Previous research has found that cigarette smokers were less likely to have used the Internet than nonsmokers [4]. However, this study used data from 2002 and 2004. As access to the Internet is a fast growing phenomenon, is it still the case that cigarette smokers are less likely to use the Internet?

The third issue, smokers’ level of interest in WATIs, is important because interventions are unlikely to have an impact on the prevalence of smoking if only a small proportion of the population will access them. To a certain extent, this question can be addressed by looking at the volume of people who already access WATIs (reviewed in [5-7]). However, there is also benefit in asking this question more directly. That is, in a representative general population sample of smokers, how many say they would be interested in WATIs and what factors are associated with this interest?

## Methods

A random digit dialing survey was conducted of 8467 respondents, 18 years and older, in Ontario, Canada. The survey employed a two-stage sampling design in which random digit dialing was used to identify eligible households and then an adult was selected within the household by choosing the resident whose birth date was closest to the date of the telephone interview. Approximately 98% of Ontario households have landline telephones. Previous research has demonstrated that telephone surveys marginally over-represent younger respondents and those with more education [8,9]. The current survey was conducted from September 2006 to the end of August 2007. The effective response rate was 51.7%. Analyses are presented using weighted data. Sample sizes are presented as unweighted data.

As part of the survey, respondents were asked their current smoking status (daily, occasional, nonsmoker), and daily smokers were asked their number of cigarettes smoked per day and time after waking to their first cigarette [10]. Daily smokers were also asked whether they would be “interested in a confidential program that you could access on the Internet, free of charge, that would allow you to check your smoking and compare it to other Canadians.” At the end of the survey, respondents were asked a series of demographic questions and questions about their use of the Internet: (1) had they used the Internet in the last year; (2) if yes, where did they use it (at home only, elsewhere only, or both); and (3) how often they used the Internet in the last year (dichotomized for this analysis into daily/almost daily versus other; never is included in the “other” category).

## Results

Of the entire sample, 17% were daily smokers, 5.8% were occasional smokers, and 77.2% classified themselves as nonsmokers. (Prevalence rates are similar to those reported for the 2006 Canadian Tobacco Use Monitoring Survey [11]). Table 1 compares demographic and Internet use characteristics between these three groups. As has been seen with other general population surveys (eg, [11]), there were systematic differences in demographic characteristics across the three smoking status groups. (These differences will not be described in detail here as they are not the purpose of this paper and because demographic differences between smokers and nonsmokers have been well described in other publications.) There were also differences in level of Internet use between smokers and nonsmokers. Smokers appeared marginally less likely than nonsmokers to have used the Internet in the last year (daily smokers = 74.5%, occasional smokers = 78.7%, nonsmokers = 81.0%;*χ* ^2^_2_ = 30.8, *P* < .001; combined sample = 79.8%). There were also significant differences in location of Internet use between smokers and nonsmokers, although the pattern of results is difficult to interpret in a meaningful fashion (*χ* ^2^_4_ = 33.3, *P* < .001). In addition, there were differences between smokers and nonsmokers in the proportion of respondents who used the Internet daily or almost daily. While 56.5% of the entire sample reported using the Internet daily, 47.8% of smokers, 56.2% of occasional smokers, and 58.5% of nonsmokers reported daily or almost daily use of the Internet (*χ* ^2^_2_ = 54.7, *P* < .001). Finally, among daily smokers, those who used the Internet daily or almost daily smoked marginally fewer cigarettes per day than those who did not use the Internet daily (mean = 14.7, SD = 7.4 vs mean = 15.7, SD = 8.1, respectively; *t*_1423_ = 2.5, *P* = .01).

**Table 1:** Demographic and Internet use characteristics among daily smokers, occasional smokers, and nonsmokers (N = 8454; 13 respondents did not provide smoking status.)

	Daily Smoker (N = 1433)	Occasional Smoker (N = 494)	Nonsmoker (N = 6527)	*P*
Age, mean (SD)	42.7 (13.8)	41.8 (15.3)	47.3 (16.7)	.001
Male, %	49.0	49.7	43.8	.001
Some post-secondary education, %	50.8	65.1	68.9	.001
Married/common law, %	59.5	59.4	70.2	.001
Full- or part-time employed, %	71.1	70.1	61.6	.001
**Family income (thousands of Can $), %**				
< 30	16.0	11.8	10.7	
30-49	15.6	14.7	13.5	
50-79	23.6	19.4	19.9	
80 or more	27.9	35.7	34.7	
Don’t know/refused	16.9	18.4	21.2	.001
Any Internet use in past 12 months, %	74.5	78.7	81.0	.001
Use Internet daily/almost daily, %	47.8	56.2	58.5	.001
**Location of Internet use, %**				
At home only	36.2	24.2	33.1	
Elsewhere only	7.8	6.8	5.1	
Both home and elsewhere	56.0	69.0	61.8	.001

### Predicting Internet Use

Table 2 displays a logistic regression predicting any Internet use in the last 12 months. Of the demographic variables, older respondents and those with family incomes less than Can $30,000 were less likely to have used the Internet. Post-secondary education, being married, and being employed were positively associated with having used the Internet in the last year. As was observed in the bivariate analyses, smoking status was also related to Internet use, with daily smokers being less likely to have used the Internet in the last year compared to other respondents (occasional and nonsmokers combined for this analysis).

**Table 2:** Logistic regression predicting respondents who did or did not use the Internet in the last year

Predictor	B (SE)	*P*
Age	−.063 (.003)	.001
Male	−.077 (.070)	.27
Some post-secondary education	1.462 (.069)	.001
Married	.293 (.078)	.001
Employed	.281 (.080)	.001
Household income less than Can $30,000	−.869 (.101)	.001
Daily cigarette smoker	−.547 (.190)	.001

### Interest in Web-Assisted Tobacco Interventions

As a measure of level of interest in WATIs, daily smokers were asked if they would be“interested in a confidential program that you could access on the Internet, free of charge, that would allow you to check your smoking and compare it to other Canadians.” Overall, 40% of daily smokers said that they would be interested in this type of WATI. Table 3 shows the demographic, smoking, and Internet use characteristics for smokers who were interested in the WATI versus those who were not. Compared to smokers who were not interested in the WATI, those who were interested were younger (*t*_1401_ = 5.9, *P* < .001), more educated (*χ* ^2^_1_ = 4.3, *P* = .04), more likely to be employed (*χ* ^2^_1_ = 11.3, *P* < .001), and probably had higher income (*χ* ^2^_4_ = 17.5, *P* = .002; note that 17% of daily smokers refused to report, or did not know, family income, making this variable difficult to interpret). Number of cigarettes smoked per day was not significantly related (*P* = .18) to interest in the WATI. However, smokers who had their first cigarette of the day within one half hour of waking were more interested in the WATI compared to those who had their first cigarette at a later time (*χ* ^2^_1_ = 11.6, *P* < .001). Finally, there was a strong relationship between level of Internet use and interest in WATI. Smokers that accessed the Internet were more interested in the WATI compared to those who did not (46% vs 20%; *χ* ^2^_1_ = 79.3, *P* < .001), or, to state it as presented in Table 3, 67% of those not interested in the WATI accessed the Internet in the past 12 months and 88% of those interested had accessed the Internet). In addition, smokers who used the Internet daily or almost daily were more likely to be interested in the WATI compared to those who used the Internet less frequently (*χ* ^2^_1_= 26.4, *P* < .001).

**Table 3:** Demographic, smoking, and Internet use characteristics for daily smokers who were interested or not interested in the WATI (N = 1424; 9 daily smokers did not provide information on their interest in WATI).

	Not Interested (N = 882)	Interested (N = 542)	*P*
Age, mean (SD)	44.3 (14.4)	40.1 (12.1)	.001
Male, %	49.4	48.5	
Some post-secondary education, %	48.7	54.4	.05
Married/common law, %	59.7	59.3	
Full- or part-time employed, %	67.9	76.8	.001
**Family income (thousands of Can $), %**			
< 30	16.8	14.3	
30-49	15.1	16.6	
50-79	21.5	27.0	
80 or more	26.7	29.5	
Don’t know/refused	19.9	12.7	.01
Cigarettes per day, mean (SD)	15.0 (7.9)	15.5 (7.6)	
Smoke within half hour of waking, %	49.9	59.3	.001
Any Internet use in past 12 months, %	66.6	87.6	.001
Use Internet daily/almost daily, %	42.5	56.5	.001
**Location of Internet use, %**			
At home only	35.1	37.4	
Elsewhere only	10.7	7.7	
Both home and elsewhere	54.2	55.0	.001

Table 4 displays the results of a logistic regression predicting interest in the WATI among daily smokers. Of the demographic characteristics, only age remained significantly related to interest in the WATI when all other variables were entered simultaneously—younger smokers were marginally more interested in the WATI compared to older smokers. Smokers who had used the Internet in the last year were more likely to be interested in the WATI compared to those who had not used the Internet. However, daily use of the Internet was not significantly related. Finally, number of cigarettes smoked per day was not related to interest in the WATI, but smokers who had their first cigarette within half an hour of waking were more likely to be interested in the WATI compared to those who had their first cigarette later in the day.

**Table 4:** Logistic regression predicting daily smoking respondents who were interested or not interested in WATI

Predictor	B (SE)	*P*
Age	−.013 (.005)	.02
Male	−.155 (.127)	.22
Some post-secondary education	−.146 (.130)	.26
Married	.152 (.132)	.25
Employed	.217 (.149)	.14
Household income less than Can $30,000	.057 (.196)	.77
Used Internet in past 12 months	1.101 (.190)	.001
Used Internet daily	.115 (.141)	.41
Cigarettes smoked per day	.014 (.009)	.14
Smoke within half hour of waking	.428 (.137)	.002

## Discussion

While many more smokers used the Internet in 2007 compared to that observed in 2002 [4], smokers were still less likely to use the Internet compared to nonsmokers. (In 2002, 65% of daily smokers in Ontario had accessed the Internet in the past 12 months [8].) However, as almost three-quarters of smokers report using the Internet, at least in Ontario, Canada, it can safely be said that the majority of smokers do not experience lack of Internet access as a barrier to using WATIs. It is likely that more smokers and nonsmokers will use the Internet in the years to come as access to the Internet is growing in all subsections of the population [12].

One issue to consider is that there does not appear to be any theoretical reason why smoking cigarettes per se is causally related to Internet access. It is likely that smoking is a marker for other demographic characteristics that are related to Internet access (eg, socioeconomic status). However, while interesting, this issue is not of practical relevance to the current paper as this study examines whether smokers have access to the Internet and not the reasons why smokers might have less access than nonsmokers.

Many smokers say that they would be interested in one type of WATI, with 40% of daily smokers saying that they would be interested in an Internet-based program that would allow them to compare their smoking to other Canadians. A clear limitation of this question as a means of assessing level of interest in WATIs is that normative comparison programs (ie, comparing own smoking to that of others) are just one type of WATI available. It is possible that smokers might be more (or less) interested in other types of WATI, just as smokers indicate variation in interest in WATIs versus other types of services (eg, telephone counseling) [13]. Another limitation is that stating an interest in WATIs on a general population telephone survey does not necessarily mean that the smoker would actually access such a program. Nevertheless, it is encouraging that so many smokers say that they would be interested in this type of WATI.

Other factors related to interest in WATIs were younger age and having used the Internet in the last year. Smokers who had their first cigarette within half an hour of waking were more interested in WATIs compared to those who waited a longer time until smoking, perhaps indicating that smokers with greater dependency are more likely to be interested in WATIs. Further research could test the relationship between level of dependence and interest in WATIs. Also of relevance would be whether smokers’ readiness to change is related to interest in WATIs. Finally, while reports of the number of smokers using WATIs are impressive [6,14], it is clear that far less than 40% of smokers are actually using them. What keeps the remaining smokers from accessing these services? This is an issue of importance for further research as WATIs take their place as an important component in tobacco cessation efforts worldwide.

## Abbreviations

WATI: Web-assisted tobacco intervention

## References

[1] Etter Jean-François (2005). Comparing the efficacy of two Internet-based, computer-tailored smoking cessation programs: a randomized trial. J Med Internet Res.

[2] Strecher Victor J, Marcus Al, Bishop Kathy, Fleisher Linda, Stengle William, Levinson Arnold, Fairclough Diane L, Wolfe Pam, Morra Marion, Davis Sharon, Warnecke Richard, Heimendinger Jerianne, Nowak Mike (2005). A randomized controlled trial of multiple tailored messages for smoking cessation among callers to the cancer information service. J Health Commun.

[3] Swartz L H G, Noell J W, Schroeder S W, Ary D V (2006). A randomised control study of a fully automated internet based smoking cessation programme. Tob Control.

[4] Cunningham John A, Selby Peter L, Kypri Kypros, Humphreys Keith N (2006). Access to the Internet among drinkers, smokers and illicit drug users: is it a barrier to the provision of interventions on the World Wide Web?. Med Inform Internet Med.

[5] Etter Jean-François (2006). Internet-based smoking cessation programs. Int J Med Inform.

[6] Etter Jean-François (2006). The internet and the industrial revolution in smoking cessation counselling. Drug Alcohol Rev.

[7] Walters Scott T, Wright Jo Anne, Shegog Ross (2006). A review of computer and Internet-based interventions for smoking behavior. Addict Behav.

[8] Adlaf EM, Ialomiteanu A (2003). CAMH Monitor Technical Guide, 2002.

[9] Trewin D, Lee G (1988). International comparisons of telephone coverage. Groves RM, Biemer PP, Lyberg LE, Massey W, Nicholls L, Waksberg J, editors. Telephone Survey Methodology.

[10] Heatherton T F, Kozlowski L T, Frecker R C, Rickert W, Robinson J (1989). Measuring the heaviness of smoking: using self-reported time to the first cigarette of the day and number of cigarettes smoked per day. Br J Addict.

[11] Health Canada (2006). Canadian Tobacco Use Monitoring Survey (CTUMS) 2006. Health Canada.

[12] United States Department of Commerce (2002). A Nation Online: How Americans Are Expanding Their Use of the Internet.

[13] Cobb Nathan K, Graham Amanda L (2006). Characterizing Internet searchers of smoking cessation information. J Med Internet Res.

[14] Cobb Nathan K, Graham Amanda L, Bock Beth C, Papandonatos George, Abrams David B (2005). Initial evaluation of a real-world Internet smoking cessation system. Nicotine Tob Res.

